# A national data linkage study to determine the association of congenital anomalies with standardised education outcomes.

**DOI:** 10.23889/ijpds.v7i3.1845

**Published:** 2022-08-25

**Authors:** Anna Rawlings, Ieuan Scanlon, Svetlana Glinianaia, Judith Rankin, Maria Loane, Joan Morris, Dan Thayer, David Tucker, Sue Jordan

**Affiliations:** 1 Swansea University; 2 Newcastle University; 3 Ulster University; 4 University of London; 5 Public Health Wales

## Objectives

Education outcomes predict life chances; however disabilities and concurrent learning difficulties are often barriers to school performance. Low educational achievement is also linked with socio-economic deprivation. We aimed to quantify the association of these factors with education outcomes by ages 12 and 17 for children in Wales (UK).

## Approach

We linked health, socio-economic and education data for children born after 1997 in Wales, registered with any one of 17 isolated congenital anomalies. Children were included if they had known education outcomes at ages 12 (n=9,223) and 17 (n=4524). A reference population comprised children born similarly but without anomalies, also having education records at 12 (n=261,827) and 17 (n=4,524).

Multinomial logistic regressions determined the likelihood of attainment in compulsory subjects by age 12 and of attaining five GCSE qualifications by age 17. We adjusted for socio-economic status by using free school meal eligibility (household deprivation) and area-based indices of deprivation.

## Results

We identified 14 and 11 isolated anomalies that reduced the likelihood of achieving educational standards by ages 12 and 17 respectively. Educational achievements were lower for children with Down syndrome, Turner syndrome, hydrocephalus and limb reduction defects, particularly at age 12, indicating that children with anomalies may catch-up with their peers. While children with anomalies were less likely to achieve five GCSEs by age 17, there was no difference in the grades of those who did so. Deprivation, particularly at household level, further reduced the likelihood of education success. At age 17, household deprivation reduced 3-fold the odds of achieving any five GCSEs grades A*-C.

## Conclusion

Children with congenital anomalies from the most deprived communities and households are at particular risk of low education achievement. To realise their potential, these children should be identified from medical records at an early age and offered targeted educational support as soon as possible and at every opportunity.

